# Acoustic recordings and modeling under seasonally varying sea ice

**DOI:** 10.1038/s41598-019-44707-0

**Published:** 2019-06-06

**Authors:** Michael D. Collins, Altan Turgut, Richard Menis, Jeffrey A. Schindall

**Affiliations:** Code 7160, Naval Research Laboratory, Washington, D.C. 20375 USA

**Keywords:** Physical oceanography, Acoustics

## Abstract

Acoustic data from the Canada Basin Acoustic Propagation Experiment are discussed. These recordings were obtained under seasonally varying sea ice to the north of Alaska during a period of 154 days. They contain signals from sources that were deployed at ranges of 17.5, 29.6, and 237.8 km and ambient sounds from marine mammals and ice-related events. After the area was covered with ice, the amplitude of receptions from the most distant source gradually decreased as scattering features on the underside of the ice developed during fracturing, drifting, ridging, and rafting events. Improvements are presented for an Arctic acoustic model that is based on the parabolic equation method, and the approach is applied to a problem in which variable ice thickness acts as a loss mechanism by scattering energy out of the waveguide. Some of the recordings have a harmonic signature that is believed to be associated with the resonances of ice floes rubbing together, but variations in the harmonics over short time scales cannot be explained in terms of the resonances of an isolated floe. This behavior may be related to the coupling of vibrations at contact points that vary during the relative motions of floes.

## Introduction

Changing conditions in the Arctic, such as diminishing sea ice^1^, is one of the factors that recently led to a renewed interest in Arctic acoustics and motivated the Canada Basin Acoustic Propagation Experiment (CANAPE). Participants from several institutions carried out the deployment phase of CANAPE during two sea trips to the north of Alaska in 2016. This paper discusses acoustic recordings that were obtained on an array of receivers that was deployed during CANAPE. These data cover a period of 154 days and include signals from sources that were deployed at ranges of 17.5, 29.6, and 237.8 km from the array and ambient sounds from sea mammals and ice-related events. Some of the recordings consist of harmonics that are believed to be associated with the resonances of ice floes^2,3^. The pronounced variations of the harmonics cannot be explained in terms of the resonances of an isolated floe; this effect may be related to the coupling of the vibrations of floes at moving contact points. Data from CANAPE are compared with results obtained with the parabolic equation method^4^, which is useful for solving large-scale wave propagation problems in which there are gradual horizontal variations in the parameters of the medium. An improved approach for handling variations in ice thickness is presented for the Arctic parabolic equation^5^, which was not available during previous studies in Arctic acoustics^6–13^. Applying this model for parameter ranges relevant to CANAPE led to a better understanding of the requirements for obtaining stable solutions.

## Methods and Materials

### Parabolic equation method

The parabolic equation method is based on the assumption that outgoing energy dominates energy that is backscattered toward the source in an environment in which horizontal variations are gradual. Due to an unmatched combination of accuracy and efficiency, this approach is often applied to large-scale wave propagation problems before it becomes practical to fully test the results using other approaches. When it was applied to a mode cut-off problem in which trapped modes in the ocean couple into beams of energy in the sediment^14^, the results were not tested initially but were later found to be in agreement with a coupled mode solution^15^. When it was applied to a problem involving refraction of acoustic waves by the zonal winds in the atmosphere of Jupiter^16^, the results were tested only in a qualitative sense using ray solutions. Accuracy is tested here for problems in Arctic acoustics using a finite-element model for directly solving the elastic wave equation^17^. It is not practical to run this model at high frequencies, but it is usually sufficient to test the accuracy of a parabolic equation model at low frequencies since (a) continuous horizontal variations in the properties of the medium become more gradual on the scale of a wavelength as frequency increases and (b) horizontal variations associated with a sloping interface do not scale with frequency.

The parabolic equation method is described here for a two-dimensional problem in an elastic medium, where *x* is the horizontal coordinate and the parameters of the medium are initially assumed to depend only on the depth *z*. The elastic wave equation may be written in the form^5^,

1 $$(L\frac{{{\rm{\partial }}}^{2}}{{\rm{\partial }}{x}^{2}}+M)(\begin{array}{c}{u}_{x}\\ w\end{array})=(\begin{array}{c}0\\ 0\end{array}),$$

where *u*_*x*_ is the horizontal derivative of the horizontal displacement and *w* is the vertical displacement. The depth operators that appear in *L* and *M* are defined by the equations,

2 $$(\lambda +2\mu )\frac{{{\rm{\partial }}}^{2}{u}_{x}}{{\rm{\partial }}{x}^{2}}+\frac{{\rm{\partial }}}{{\rm{\partial }}z}(\mu \frac{{\rm{\partial }}{u}_{x}}{{\rm{\partial }}z})+\rho {\omega }^{2}{u}_{x}+(\lambda +\mu )\frac{{{\rm{\partial }}}^{3}w}{{\rm{\partial }}{x}^{2}{\rm{\partial }}z}+\frac{{\rm{\partial }}\mu }{{\rm{\partial }}z}\frac{{{\rm{\partial }}}^{2}w}{{\rm{\partial }}{x}^{2}}=0,$$

3 $$\mu \frac{{{\rm{\partial }}}^{2}w}{{\rm{\partial }}{x}^{2}}+\frac{{\rm{\partial }}}{{\rm{\partial }}z}((\lambda +2\mu )\frac{{\rm{\partial }}w}{{\rm{\partial }}z})+\rho {\omega }^{2}w+(\lambda +\mu )\frac{{\rm{\partial }}{u}_{x}}{{\rm{\partial }}z}+\frac{{\rm{\partial }}\lambda }{{\rm{\partial }}z}{u}_{x}=0,$$

where *ω* is the angular frequency, $\rho$ is the density, the Lamé parameters *λ* and are defined by $\rho {c}_{p}^{2}=\lambda +2\mu$ and $\rho {c}_{s}^{2}=\mu$, and *c*_*p*_ and *c*_*s*_ are the compressional and shear wave speeds. The compressional and shear attenuations ${\beta }_{p}$ and ${\beta }_{s}$ (in decibels per wavelength) are taken into account by allowing the wave speeds to be complex.

For this non-conventional set of dependent variables, there is no $\partial /\partial x$ term in Eq. (1), which may be factored as follows,

4 $$(\frac{{\rm{\partial }}}{{\rm{\partial }}x}+i{T}^{1/2})(\frac{{\rm{\partial }}}{{\rm{\partial }}x}-i{T}^{1/2})(\begin{array}{c}{u}_{x}\\ w\end{array})=(\begin{array}{c}0\\ 0\end{array}),$$

where $T={L}^{-1}M$. The factors in Eq. (4) correspond to energy that is outgoing and incoming in the horizontal direction. The parabolic equation method is based on assuming that outgoing energy dominates energy that is backscattered in the horizontal direction and deriving solutions based on the parabolic wave equation,

5 $$\frac{{\rm{\partial }}}{{\rm{\partial }}x}(\begin{array}{c}{u}_{x}\\ w\end{array})=i{T}^{1/2}(\begin{array}{c}{u}_{x}\\ w\end{array}).$$

As discussed in^5^ and references therein, solutions based on Eq. (5) may be obtained using approaches for approximating the square root of the operator, the numerical implementation of the differential operators (including interface and boundary conditions), generating an initial condition, and handling horizontal variations in the medium that are sufficiently gradual so that backscattered energy is negligible relative to outgoing energy. Going from Eq. (1) to Eq. (5) often results in reductions in computation times of several orders of magnitude and makes it practical to solve large-scale problems of interest in ocean acoustics and seismology. This gain in efficiency is often achieved with no significant loss in accuracy when outgoing energy dominates and the square root of the operator is approximated using a higher-order rational function. The gain in efficiency is non-linear in frequency, and finite-element models for directly solving Eq. (1) are rarely used in ocean acoustics^4^.

### Sources and receivers

On August 26, Scripps Institution of Oceanography deployed a 225–325 Hz source 175 m below the surface in 3852 m of water at 74.3018°N 153.9499°W. The Naval Research Laboratory deployed the other hardware discussed here. On October 21, a source was deployed 192 m below the surface in 317.5 m of water at 72.8342°N 158.6625°W; it transmitted 890 Hz and 2.5 kHz cw tones, upsweeps in the 700–1100 Hz and 1.5–4 kHz bands, and m-sequences centered at 900 Hz and 2.5 kHz. On October 22, a source was deployed 99 m below the surface in 149 m of water at 72.5638°N 158.2155°W; it transmitted 910 Hz and 2.55 kHz cw tones, downsweeps in the 700–1100 Hz and 1.5–4 kHz bands, and m-sequences centered at 900 Hz and 2.5 kHz. On October 24, a 500 kHz upward-looking sonar for measuring ice draft was deployed 27 m below the surface in 166 m of water at 72.6492°N 158.5423°W. The 64-element array of receivers has a 54-element rectangular subarray (nine vertical by six horizontal), an additional ten elements in the lower horizontal subarray, and a receiver spacing of 0.432 m in both directions. On October 19, the array was deployed in 151.2 m of water at 72.7054°N 158.9691°W, with the upper horizontal subarray 93 m below the surface. Acoustic data were recorded on each receiver at 24 bits and a sampling rate of 12 kHz for 45 minutes every four hours until the batteries ran out after March 22, 2017. The array and its data were recovered on September 3, 2017. This data set represents a broad range of spatial, frequency, and temporal sampling, but the initial study presented here focuses on temporal variations and ambient sounds, for which it is sufficient to consider data from a single receiver.

## Results

### Extending the arctic parabolic equation to higher frequencies

Horizontal variations in the environment may be handled by approximating in terms of a series of regions in which the elastic parameters depend only on depth. In each region, the factorization in Eq. (4) is exact and approximations based on Eq. (5) may be used to propagate the field through each region. All that remains is to specify conditions for estimating transmitted fields across the vertical interfaces between regions. Various conditions have been proposed and found to provide accurate solutions for problems involving sloping interfaces and boundaries^18–20^. For some cases, an approach that was proposed for handling a sloping fluid-solid interface requires the introduction of an artificial intermediate region and relatively fine sampling in the computational grid^19^. The approach presented here provides comparable accuracy but greater efficiency, which can be a significant advantage at relatively high frequencies.

In the exact solution, the horizontal displacement, vertical displacement, normal stress ${\sigma }_{xx}$, and tangential stress ${\sigma }_{xz}$ are conserved across a vertical interface, where

6 $$(\begin{array}{c}{\sigma }_{xx}\\ w\end{array})=R(\begin{array}{c}{u}_{x}\\ w\end{array}),$$

7 $$(\begin{array}{c}u\\ -{\sigma }_{xz}\end{array})=-\,iS{T}^{-1/2}(\begin{array}{c}{u}_{x}\\ w\end{array}),$$

8 $$R=(\begin{array}{cc}\lambda +2\mu  & \lambda \frac{{\rm{\partial }}}{{\rm{\partial }}z}\\ 0 & 1\end{array}),$$

9 $$S=(\begin{array}{cc}1 & 0\\ \lambda \frac{{\rm{\partial }}}{{\rm{\partial }}z}+\frac{{\rm{\partial }}\lambda }{{\rm{\partial }}z} & \frac{{\rm{\partial }}}{{\rm{\partial }}z}(\lambda +2\mu )\frac{{\rm{\partial }}}{{\rm{\partial }}z}+\rho {\omega }^{2}\end{array}).$$

When backscattered energy is completely neglected in an outgoing solution, it is not possible to conserve all four quantities, but it is possible to conserve two of the quantities by applying the condition,

10 $${S}_{B}{T}_{B}^{-1/2}{(\begin{array}{c}{u}_{x}\\ w\end{array})}_{t}={S}_{A}{T}_{A}^{-1/2}{(\begin{array}{c}{u}_{x}\\ w\end{array})}_{i},$$

where the subscripts *i* and *t* denote the incident and transmitted quantities and the subscripts *A* and *B* denote evaluation on the incident and transmitted sides of the vertical interface. Conserving half of the quantities at a vertical interface may not produce accurate solutions, but it is possible to obtain accurate solutions for many problems involving sloping interfaces between ice and water with the condition,

11 $${\rho }_{B}^{1/2}{S}_{B}{T}_{B}^{-1/2}{(\begin{array}{c}{u}_{x}\\ w\end{array})}_{t}={\rho }_{A}^{1/2}{S}_{A}{T}_{A}^{-1/2}{(\begin{array}{c}{u}_{x}\\ w\end{array})}_{i},$$

which approximately reduces to a condition for conserving energy^21^ for the acoustic case $\mu =0$. It would exactly reduce to the condition for conserving energy if the operator ${T}^{-1/2}$ were replaced with ${T}^{-3/4}$ in Eq. (11), but in that case the condition ${\sigma }_{xz}=0$ would not hold on the fluid-solid parts of vertical interfaces. This condition may be implemented for both the upslope and the downslope cases (which are mathematically distinct) without the complications associated with introducing an artificial intermediate region^19^.

**Figure 1:**
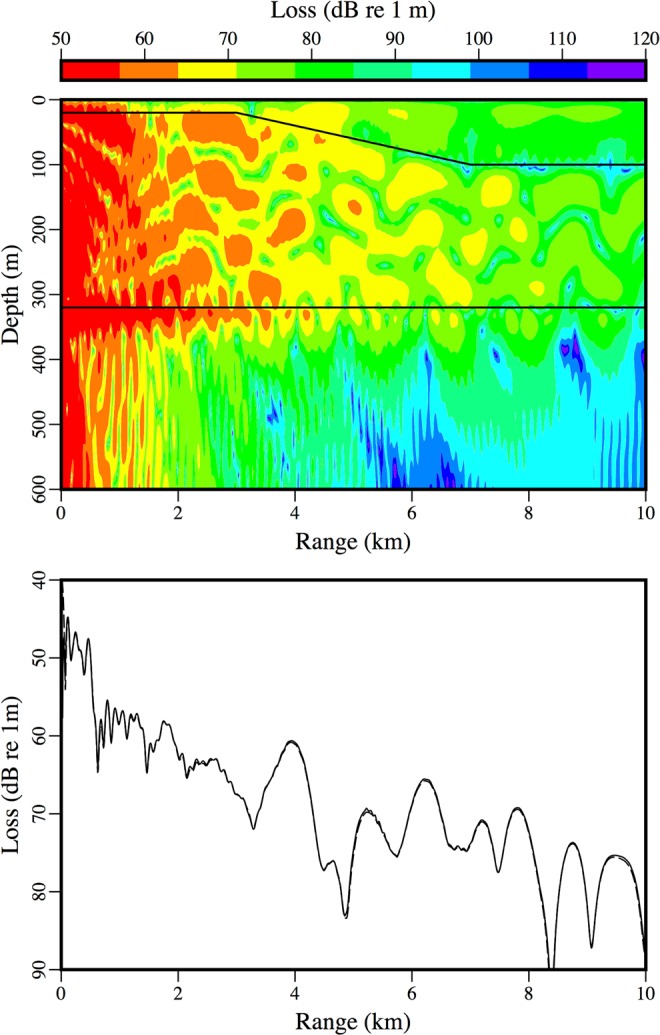
An example involving a downward sloping ice-water interface. The parabolic equation solution (solid curve) is in agreement with the finite-element solution (dashed curve).

**Figure 2:**
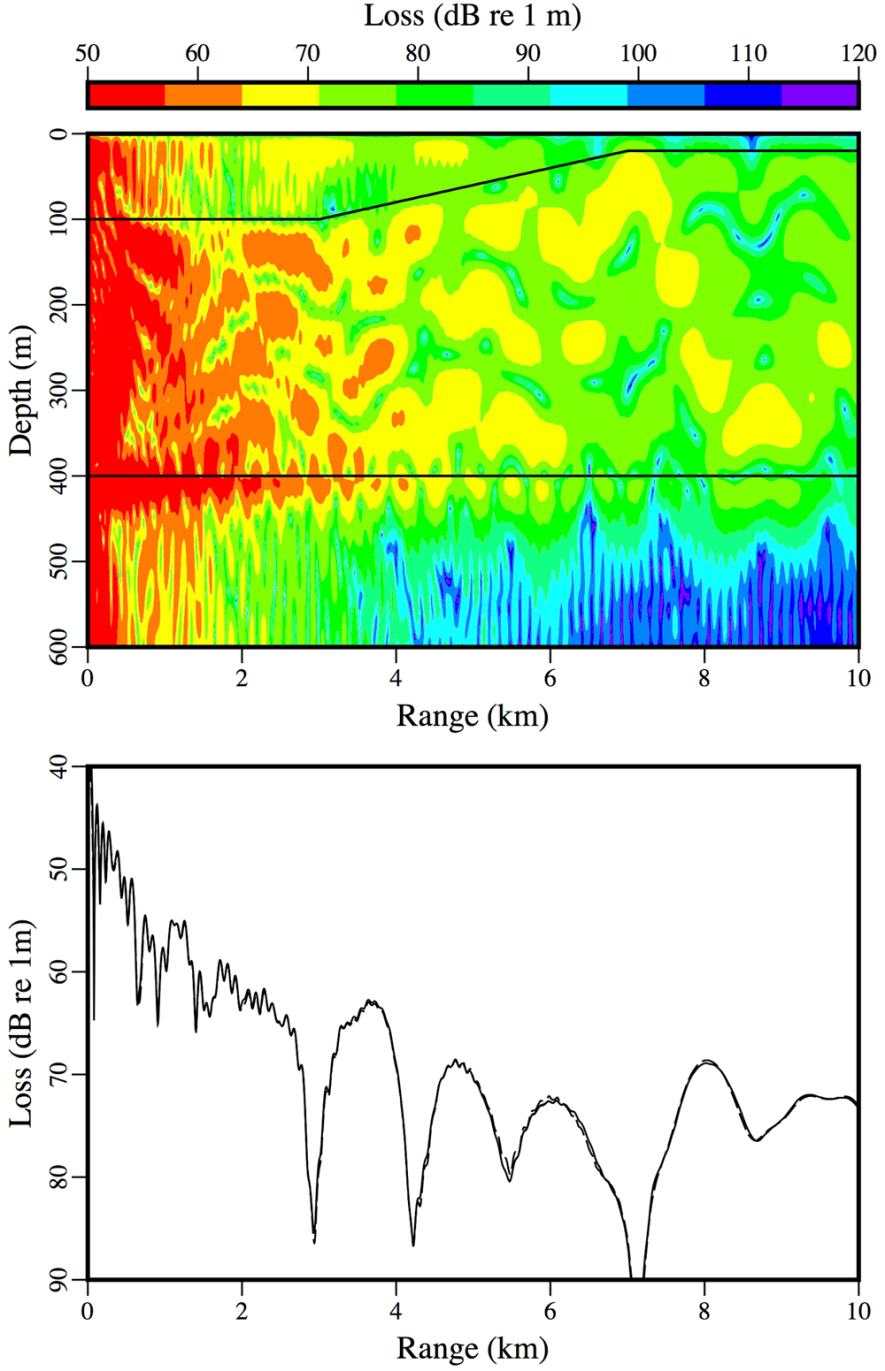
An example involving an upward sloping ice-water interface. The parabolic equation solution (solid curve) is in agreement with the finite-element solution (dashed curve).

The accuracy of the approximate condition for conserving energy in Eq. (11) is illustrated here for examples involving sloping ice-water interfaces^19^. For these problems, a 25 Hz source is located in a water column in which *c* = 1500 m/s; the parameters of the ice are *c*_*p*_ = 3500 m/s, *c*_*s*_ = 1750 m/s, $\rho$ = 0.9 g/cm^3^, ${\beta }_{p}=0.1$, and ${\beta }_{s}=0.2$; and the parameters in the sediment are *c*_*p*_ = 3400 m/s, *c*_*s*_ = 1700 m/s, $\rho$ = 2.5 g/cm^3^ ${\beta }_{p}=0.2$, and ${\beta }_{s}=0.4$. The water column is 300 m deep at the source, which is 280 m below the ice. For the downslope case, the thickness of the ice is 20 m for *r* < 3 km, 100 m for *r* > 7 km, and linearly increasing between these values for 3 km < *r* < 7 km. For the upslope case, the thickness of the ice is 100 m for *r* < 3 km, 20 m for *r* > 7 km, and linearly decreasing between these values for 3 km < *r* < 7 km. In the comparisons with a finite-element solution^17^ in Figs 1 and 2, the solutions obtained using Eq. (11) are as accurate as the solutions presented in^19^, which were obtained using an approach that is less efficient.

In applying the Arctic parabolic equation to frequencies relevant to CANAPE, it was found that a stability issue (which had apparently been overlooked previously) exists for some ranges of the parameters. The operator square root in Eq. (5) may be approximated with an expansion of the form,

12 $${T}^{1/2}={k}_{0}{(1+X)}^{1/2}\cong {k}_{0}+{k}_{0}\sum _{j=1}^{n}\frac{{a}_{j,n}X}{1+{b}_{j,n}X},$$

where *X* is defined by Eq. (12), the constant *k*_0_ is a representative wave number, and various choices for the coefficients have been proposed. Stable solutions may be obtained for many elastic wave propagation problems by using the rotated coefficients^22^,

13 $${a}_{j,n}=\frac{{e}^{-i\theta /2}{\mathop{a}\limits^{ \sim }}_{j,n}}{{[1+{\mathop{b}\limits^{ \sim }}_{j,n}({e}^{-i\theta }-1)]}^{2}},$$

14 $${b}_{j,n}=\frac{{e}^{-i\theta }{\mathop{b}\limits^{ \sim }}_{j,n}}{1+{\mathop{b}\limits^{ \sim }}_{j,n}({e}^{-i\theta }-1)},$$

15 $${\mathop{a}\limits^{ \sim }}_{j,n}=\frac{2}{2n+1}{\sin }^{2}(\frac{j\pi }{2n+1}),$$

16 $${\mathop{b}\limits^{ \sim }}_{j,n}={\cos }^{2}(\frac{j\pi }{2n+1}),$$

which are obtained by rotating the branch cut of the square root function by the angle $\theta$. The case $\theta =0$ corresponds to the coefficients of the Padé approximation in Eqs (15) and (16) ^23^, which do not provide stable solutions for problems involving shear waves^24^.

**Figure 3:**
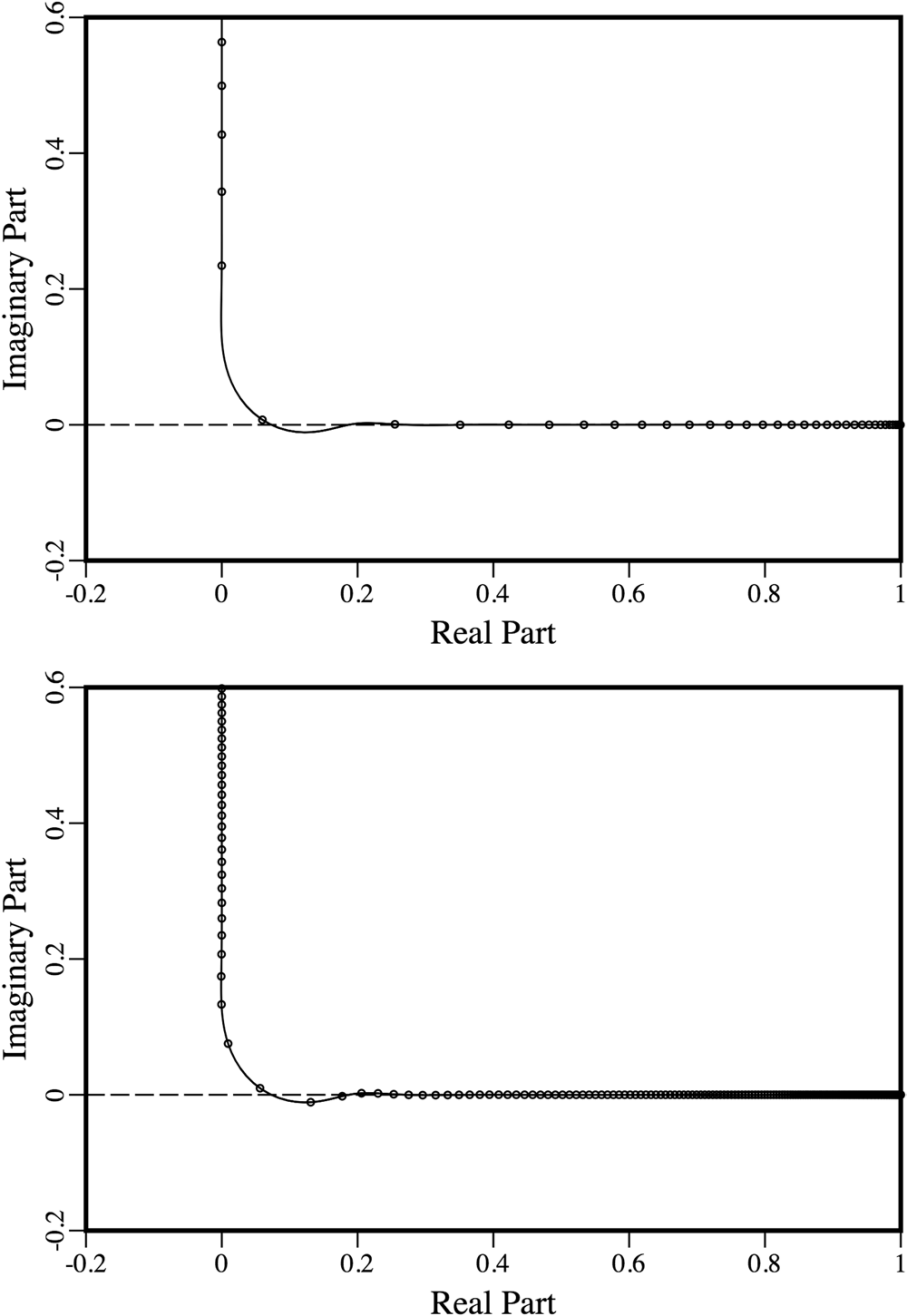
Mappings of the eigenvalues (which are shown as small circles) by rotated rational approximations. At 50 Hz (top) all of the eigenvalues are mapped on or above the real axis. At 250 Hz, a few of the eigenvalues are mapped below the real axis.

The exact square root function maps $(\,-\,\infty ,-\,1)$ to the imaginary axis and maps $(\,-\,1,\infty )$ to the real axis. Parts of both of these intervals may be handled properly with the rotated coefficients, but all rational approximations break down in a neighborhood of the branch point *X* = −1, as can be seen from the example in Fig. 3. The solid curve corresponds to the image of the real axis under the rotated rational approximation for the case *n* = 10 and $\theta =110^\circ$. Parts of the curve appear to coincide with the real and imaginary axes (within the resolution of the graph), but a short segment dips slightly below the real axis. The small circles correspond to the images of eigenvalues associated with the normal modes^4^ of an acoustics problem in a 500 m thick homogeneous waveguide, where the sound speed is 1500 m/s and the condition *p* = 0 applies at the top and bottom boundaries. At 50 Hz, all of the eigenvalues are mapped on or above the real axis, which is required for stability. The eigenvalues are more densely spaced at 250 Hz, and two of them are mapped slightly below the real axis. These eigenvalues are the cause of the stability problem, and they correspond to nearly vertical propagation. Stable solutions may be obtained by introducing attenuation that affects these eigenvalues but does not have a significant affect on eigenvalues that correspond to the propagating modes. For the examples presented here, stable parabolic equation solutions were obtained by allowing the attenuation to artificially increase deep within the sediment.

### Acoustic recordings

**Figure 4:**
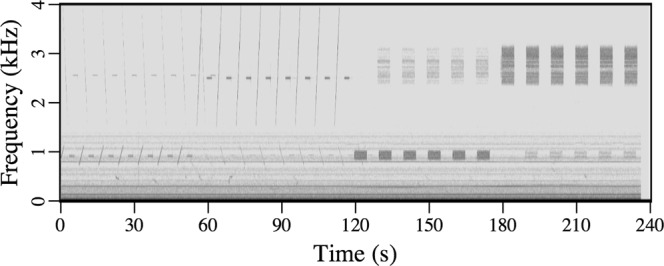
Spectrogram of Audio S1, which shows the signals received from the nearby sources.

**Figure 5:**
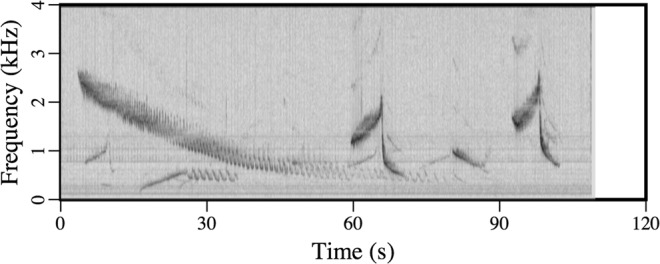
Spectrogram of Audio S2, which contains vocalizations of a Bearded Seal.

**Figure 6:**
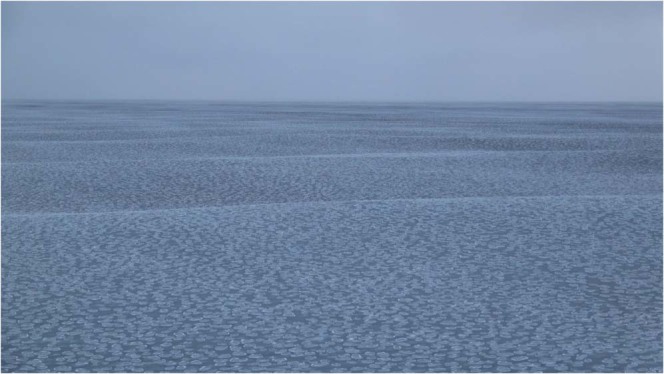
Pancake ice that began to form after the sources and receivers were deployed. Surface waves corresponding to short wavelengths are filtered out by this type of ice, as can be seen in Movie S1.

All of the acoustic recordings discussed here were obtained in 2017 on one of the end receivers of the top row of the array. All of the signal types that were transmitted by the two nearby sources are audible in Audio S1 (January 14). In the spectrogram of this recording in Fig. 4, the signals from the closer source (which can be distinguished by the fact that some of them are upsweeps) appear more intense. There are many recordings of marine mammals in the data, including a Bearded Seal (*Erignathus barbatus*) in Audio S2 (February 9). The spectrogram of this recording in Fig. 5 has characteristics in common with a spectrogram of vocalizations of this species in^25^. The area was free of ice until near the end of the second sea trip. An early stage of ice appears in the image in Fig. 6 and the video footage in Movie S1, which were obtained near 75°N 150°W on October 29. The many recordings of ice-related events in the data include stressing and fracturing in Audio S3 (February 15), numerous cracking events in Audio S4 (February 11), and strong ice breaking events in Audio S5 (February 19). Marine mammals and signals from the sources are also audible in these recordings.

**Figure 7:**
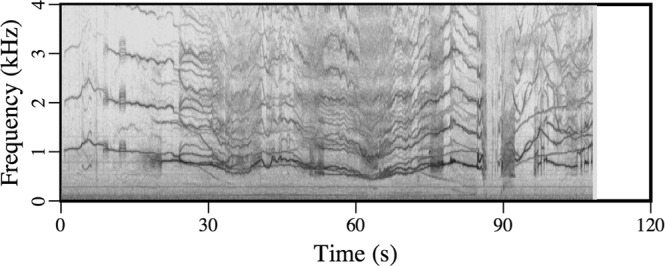
Spectrogram of sounds that are generated when moving ice floes rub together.

Some sounds that originate in the ice, such as the ones in Audio S6 (February 11), are suggestive of marine mammal vocalizations. The spectrogram of this recording in Fig. 7 has a signature of harmonics that is believed to be associated with the resonances of moving ice floes that rub against each other^2,3^. Only odd harmonics should be excited according to an analysis based on *SH* waves in plates of ice^2^, but even and odd harmonics are excited in the recording in Fig. 7. The coupling of *SH* modes to acoustic modes in the water should be relatively weak, but the received signal was strong for this event. The variations of the harmonics in Fig. 7 cannot be explained in terms of the resonances of an isolated floe, which would be expected to vary on a much larger time scale (as the size and shape of the floe vary). This effect could be related to the coupling of vibrations between floes at contact points, which vary constantly as floes slide past each other. If this hypothesis is valid, it may explain features that appear near 100 s and 1 kHz in Fig. 7; the discontinuous jumps between harmonics could be due to discontinuous jumps between contact points. Variations over short time scales could be related to temporal variations in the ambient stresses and deformations of the ice and trapped air under the ice as the floes slide past each other.

**Figure 8:**
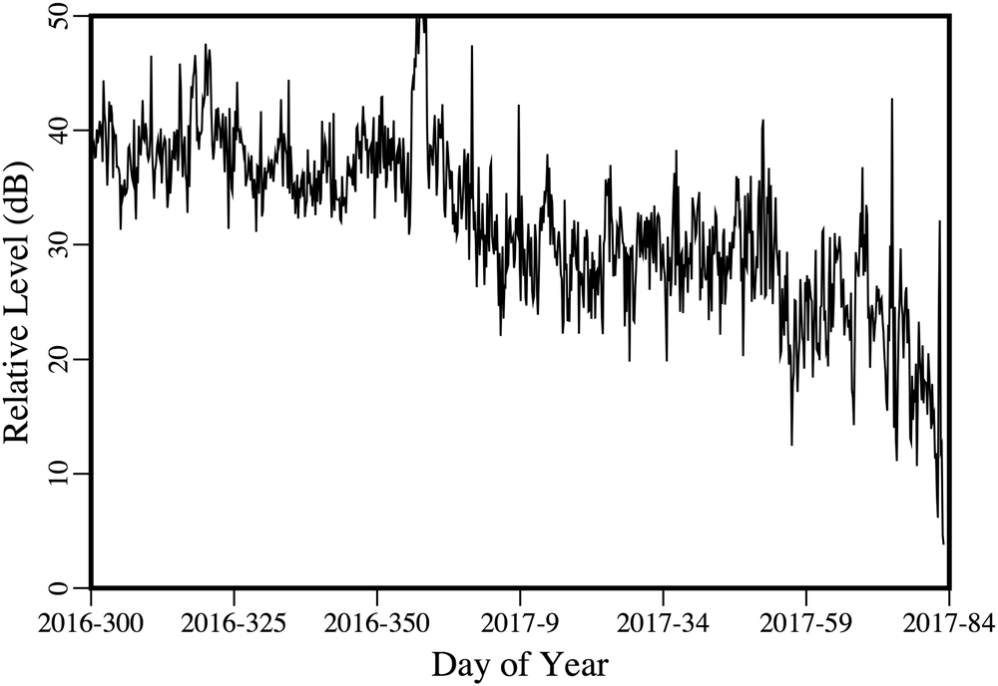
Peak intensity of the received signal from the distant source, which decreases as scattering features develop on the underside of the ice.

**Figure 9:**
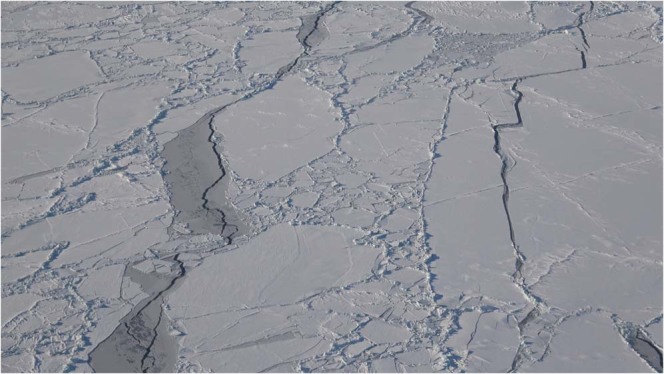
A photo that shows evidence of numerous fracturing and ridging events.

**Figure 10:**
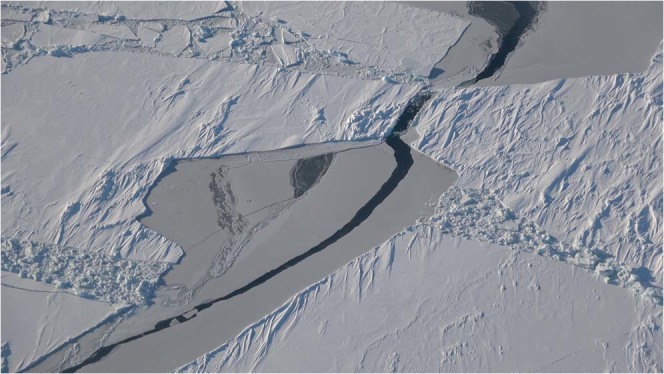
A photo that shows a large fracture that was followed by drifting.

**Figure 11:**
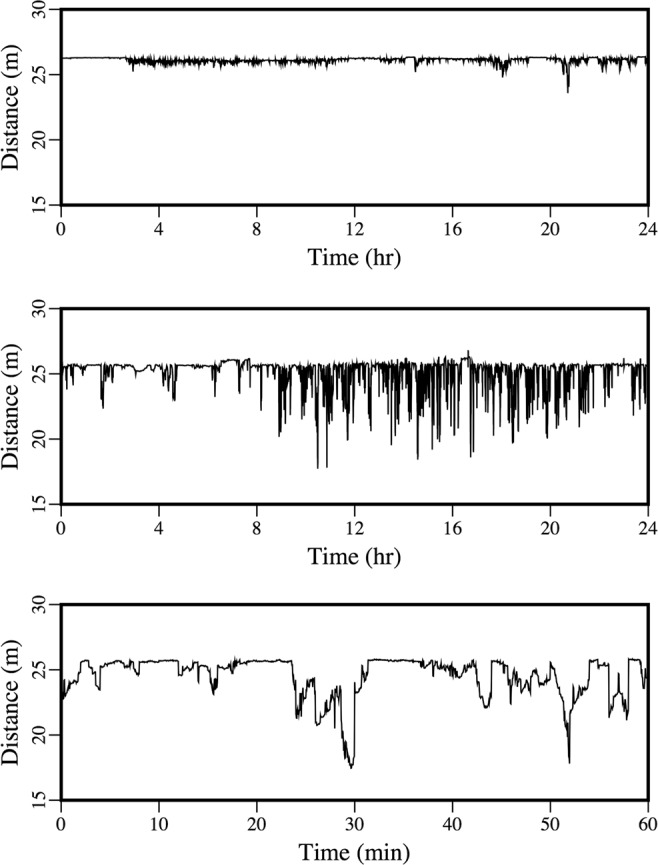
Ice profile data that show keels that drifted over the upward-looking sonar on November 19 (top) and February 2 (middle and bottom).

**Figure 12:**
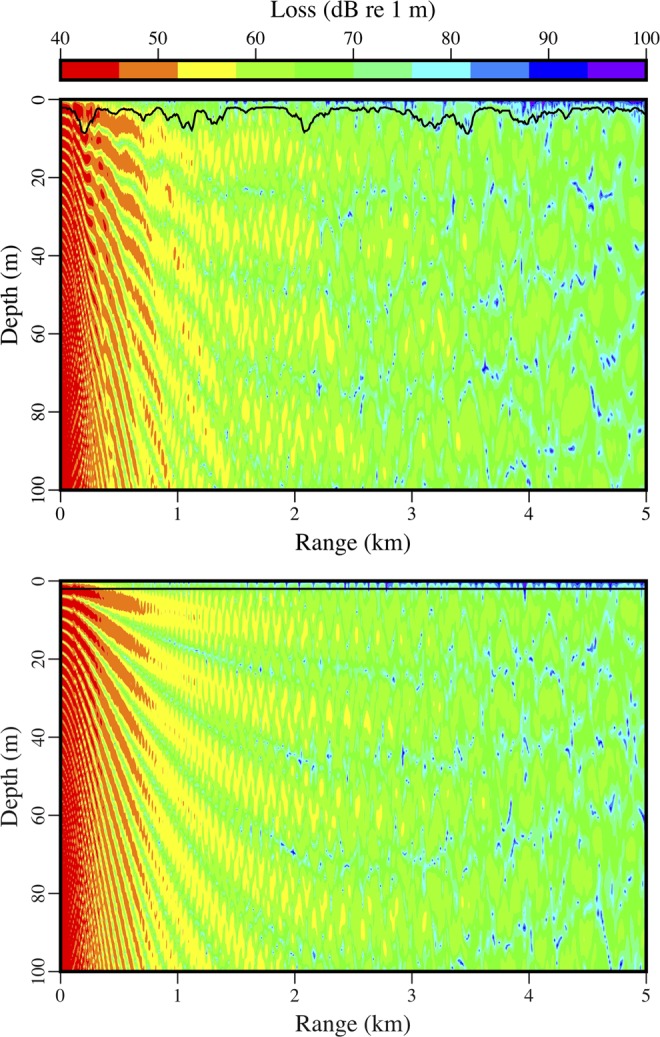
Close-up view of the acoustic field in waveguides with keels (top) and without keels (bottom). The ice-water interface is shown in both cases.

**Figure 13:**
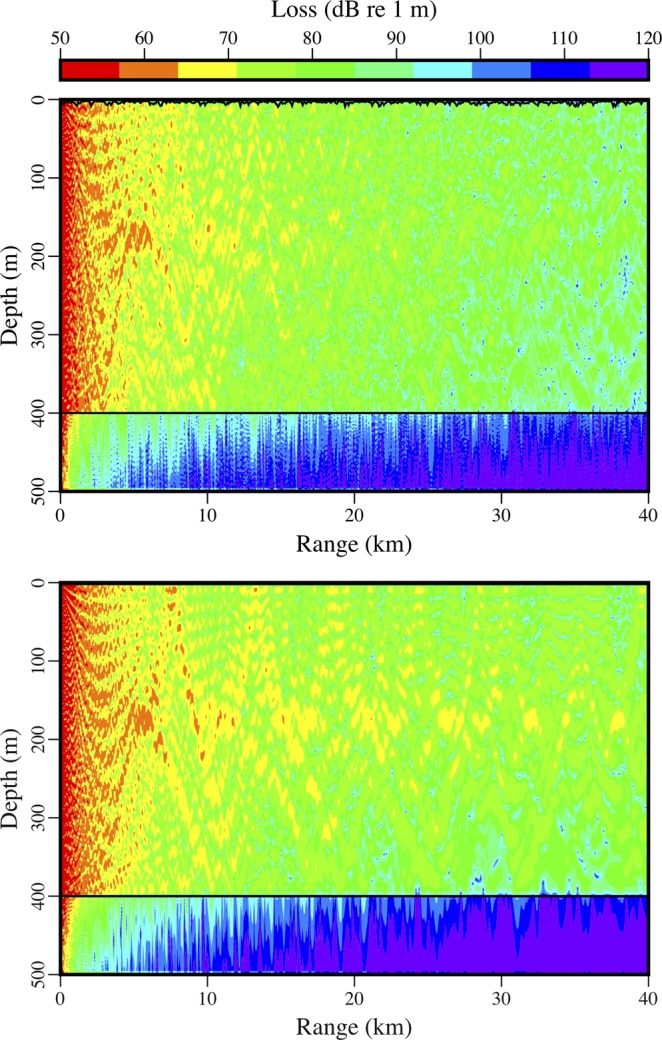
Wide view of the acoustic field in waveguides with keels (top) and without keels (bottom). Due to scattering of energy out of the waveguide, the case with keels shows less energy in the water column but more energy in the sediment.

**Figure 14:**
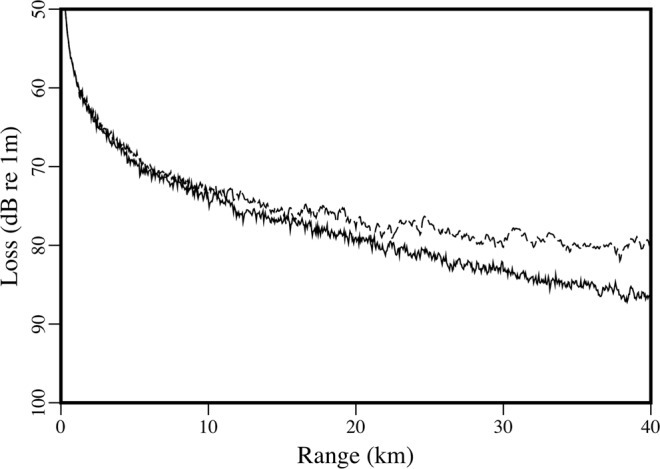
Transmission loss averaged over the entire water column at each range. Comparison for waveguides with keels (solid curve) and without keels (dashed curve).

Appearing in Fig. 8 is the variability over time of the peak amplitude of the received signal from the distant source. There were periods of strong noise, but a downward trend in amplitude began early in 2017. By that point, the area had already been covered with ice for more than a month, but it takes time for keels and other features under the ice (which tend to scatter energy out of the ocean acoustic waveguide) to develop during fracturing, drifting, rafting, and ridging^26–30^. Evidence of these processes appears in the images in Figs 9 and 10, which were obtained during a helicopter flight from Barrow to the vicinity of the array on March 18, 2017. The evolution of under-ice features with time is evident in the upward-looking sonar data appearing in Fig. 11. In order to qualitatively illustrate what is believed to be the primary loss mechanism that affects the curve in Fig. 8, the parabolic equation method is applied to a problem involving an ice profile that was generated using a five-parameter stochastic model^31^, with a mean draft of 3.5 m, rms topographic variation of 1.38 m, characteristic length of 100 m, fractal dimension of 1.5, and normalized skewness of 1.41. The speed of sound in the water column is a linear function of depth, with *c* = 1430 m/s at *z* = 0 (the surface of the ice) and *c* = 1460 m/s at *z* = 400 m. The parameters of the ice are *c*_*p*_ = 3500 m/s, *c*_*s*_ = 1750 m/s, $\rho$ = 0.9 g/cm^3^, ${\beta }_{p}=0.1$, and ${\beta }_{s}=0.2$. Below a fluid-solid interface at $z=400{\rm{m}}$, the parameters of the sediment are *c*_*p*_ = 1700 m/s, *c*_*s*_ = 800 m/s, $\rho$ = 1.5 g/cm^3^, ${\beta }_{p}=0.5$, and ${\beta }_{s}=0.5$. A 250 Hz source is located at *z* = 175 m. Appearing in Figs 12–14 are comparisons of parabolic equation solutions of this problem and a similar problem for which the ice has a constant thickness of 2 m. The under-ice features cause a significant amount of energy to scatter out of the waveguide.

## Discussion

Applications to CANAPE data motivated improvements to the Arctic parabolic equation model that are useful at higher frequencies than had been considered previously. The accuracy of an approximate energy-conservation condition for handling variable ice thickness is comparable to the accuracy of an approach that requires the introduction of an artificial intermediate material and fine sampling in the numerical grid. Some rotated rational approximations of the square root function map a short segment of the real axis below the real axis in the complex plane, and this can cause stability issues when the ocean depth is sufficiently large relative to a wavelength. The Arctic parabolic equation was used to illustrate how scattering from under-ice features acts as a loss mechanism. The received signal from a distant source began to lose intensity well after the area was covered with ice, apparently due to the time it takes for under-ice features to develop during fracturing, drifting, ridging, and rafting processes.

The array of receivers recorded for 154 days and captured many sounds from marine mammals and ice-related events. A mechanism was proposed for variations of harmonics that were previously identified with the resonances of ice floes rubbing together. The resonance frequencies of an isolated floe cannot be used to explain this behavior. Since the vibrations of two floes in contact are coupled, the resonance frequencies should vary as the contact points vary during the relative motion of the floes. This study involved data from only one receiver. By processing data from multiple receivers, it should be possible to determine the locations of ice fracturing events, possibly even for cases in which a developing fracture behaves as a moving acoustic source^32^. Due to the weak coupling of sounds from ice-related events into propagating modes^33^ and attenuation associated with scattering from under-ice features, distant ice-fracturing events (which would be received as a series of dispersed arrivals) could be detected at a lower rate than would be expected based simply on geometry.

## Supplementary information

Audio S1

Audio S2

Audio S3

Audio S4

Audio S5

Audio S6

Movie S1
